# Multifunction-oriented high-mobility polymer semiconductors

**DOI:** 10.1093/nsr/nwad253

**Published:** 2023-09-23

**Authors:** Mingliang Zhu, Yunlong Guo, Yunqi Liu

**Affiliations:** Beijing National Laboratory for Molecular Sciences, Organic Solids Laboratory, Institute of Chemistry, Chinese Academy of Sciences, China; School of Chemical Sciences, University of Chinese Academy of Sciences, China; Beijing National Laboratory for Molecular Sciences, Organic Solids Laboratory, Institute of Chemistry, Chinese Academy of Sciences, China; School of Chemical Sciences, University of Chinese Academy of Sciences, China; Beijing National Laboratory for Molecular Sciences, Organic Solids Laboratory, Institute of Chemistry, Chinese Academy of Sciences, China; School of Chemical Sciences, University of Chinese Academy of Sciences, China

## Abstract

Recent progress in multifunction-oriented high-mobility polymer semiconductors is profiled, with current challenges and future directions proposed in this perspective.

The discovery of conductive polyacetylene has initiated interdisciplinary research into condensed matter physics and synthetic chemistry in organic electronics. Unlike their inorganic and small-molecule counterparts, polymer semiconductors possess the inherent advantages of facile solution processing, large-area manufacturing, excellent mechanical strength and intrinsic flexibility. To date, they have delivered over-the-benchmark mobility of 10 cm^2^ V^–1^ s^–1^, while device technology and controllable doping/blending further enable performance enhancement and ambient stability [1]. Beyond high mobility, integration of additional mechanical (stretchability), optical (luminescence and photopatterning) and thermal (thermoelectric conversion) properties contributes to multimode fusion and intelligent manufacturing, wherein adjustable π-conjugation components can functionalize as-prepared materials towards diverse applications (Fig. 1). In this perspective, we will summarize recent progress in functional high-mobility polymer semiconductors, with current challenges and future directions proposed.

**Figure 1.**
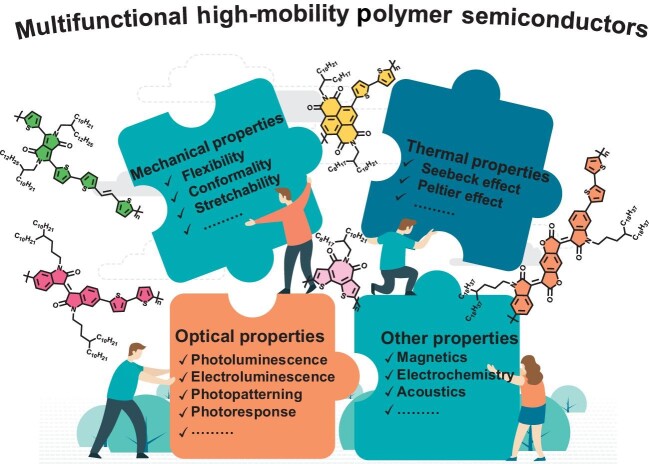
Integration of mechanical, optical, thermal or other properties towards multifunctional high-mobility polymer semiconductors.

## HIGH MOBILITY INTEGRATED WITH HIGH STRETCHABILITY

Next-generation wearable and implantable electronic devices require skin-like stretchable semiconducting materials. In contrast to the complex manufacturing of structure engineering and long-term metastable-state instability in the blending system, intrinsically stretchable polymer semiconductors possess an innate strain-accommodating capability and excellent mobility retention under strain.

Bao *et al*. initially introduced amide-type flexible-segment 2,6-pyridine dicarboxamide (PDCA) into an all-conjugated polymer backbone with the aim of efficient stress dissipation via dynamic non-covalent crosslinking [2]. They achieved a desirable balance between electrical and mechanical properties at the modification ratio of 10%, and this material exhibited mobility up to 1.32 cm^2^ V^–1^ s^–1^ initially and preserved 1.12 cm^2^ V^–1^ s^–1^ at 100% strain along the direction perpendicular to the strain, delivering a maximum mobility of 0.6 cm^2^ V^–1^ s^–1^ in fully stretchable transistors. In particular, a self-healing ability was also observed, with filed-effect mobilities fully recovered after heating or solvent annealing. Taking advantage of coordination interactions between Fe(III) ion and PDCA, the researchers blended this polymer semiconductor with an insulating poly(dimethylsiloxanealt-2,6-pyridinedicarbozamine) elastomer to confer strain sensitivity, stretchability and self-healing capabilities onto the target skin-like active semiconductor [3]. After partial metal-ligand bonding exchange and rational controlling of bicomponent percolation, blending films exhibited high sensitivity with an unprecedented gauge factor of 5.75 × 10^5^ at 100% strain in stretchable transistors, yielding excellent stretchability over 1300% and room-temperature self-healing.

Without breaking intrachain conjugation or weakening interchain order, Zhang *et al*. incorporated different non-centrosymmetric spiro-fluorine units into the reference conjugated polymer [4]. Randomly distributed spiro-fluorene units undermined film crystallinity, reduced bulky side chains, and shortened lamellar and *π*−*π* stacking distances. In particular, the cyclopropane-inserted spiro-fluorene unit endowed the designed polymer with a low elastic modulus of 83.7 MPa, and record-high mobility of 3.1 cm^2^ V^–1^ s^–1^ at 150% strain and 1.4 cm^2^ V^–1^ s^–1^ after 1000 stretching–releasing cycles at 50% strain. Generally, whether a conjugation interruption is involved or not, these materials have been established as a trade-off between film crystallinity and mechanical stretchability, but the essential issue of (micro)structure–property relationship is ambiguous and high-mobility stretchable n-type semiconductors are nearly blank. Inspired by microscopic morphology, Facchetti *et al*. reported an amphiphilic semiconducting polymer combined with uniform honeycomb-like microstructure, realizing intrinsically stretchable and electrochemically redox-free organic electrochemical transistors (OECTs) [5]. Through biaxial pre-stretching to stabilize electronic-ionic transport, OECTs with initial normalized transconductance of 16.16 S cm^–1^ delivered excellent biaxial stretchability (up to 140%), mechanical durability (10^4^ stretching–releasing cycles at 30% strain) and stable signal amplification for electrocardiogram and synapse response under strain.

## HIGH MOBILITY INTEGRATED WITH STRONG LUMINESCENCE

Revolutionary miniatured optoelectronic devices, including electrically pumped polymer lasers, organic light-emitting transistors (OLETs) and intrinsic drive-integrated displays, require integration of high mobility and strong solid-state luminescence. Generally, dense π-stacking and delocalized electron-cloud distribution facilitate carrier transport, whereas intense emission often requires attenuated intermolecular packing and localized excitons.

To simultaneously improve mobility and fluorescence, Sirringhaus *et al*. designed a class of near-amorphous ladder-type conjugated polymers with gradually extended rigid backbones and promoted close-crossing points for low energetic disorder and three-dimensional carrier-percolation pathways [6]. Interchain high-degree short contacts and an appropriate energy level of 2.0 eV contributed to a high mobility exceeding 2.4 cm^2^ V^–1^ s^–1^ and a luminescence quantum yield over 15% enabled by pinned internal charge transfer. Taking intramolecular electronic coupling into account, Yu *et al*. designed a ladder-type copolymer with enhanced crystallinity and backbone coplanarity based on the weak donor-acceptor-coupling strategy, which adopted ideal J-aggregation with a large π−π stacking distance of 4.0 Å [7]. The polymer exhibited a high fluorescence quantum yield of 77%, and ambipolar characteristics, with hole and electron mobilities of 1.26 × 10^−3^ and 3.53 × 10^−4^ cm^2^ V^–1^ s^–1^, respectively, delivering a desirable external quantum efficiency of 5.3% with electroluminescence intensity of 414 nW in multi-layered OLET devices.

Different from quasi-two-dimensional horizontal transport, vertical devices are suitable for the vigorous requirements of even carrier injection, transport and recombination luminescence. Liu *et al*. developed a self-assembled three-dimensional penetrating nano-network comprising equal-mass super-yellow poly(*p*-phenylene vinylene) and polyacrylonitrile, realizing intrinsic stretchability over 100% and 5–6-fold mobility enhancement with photoluminescence well maintained [8]. Assisted by a highly efficient stretchable electron-injection layer, intrinsically stretchable polymer light-emitting diodes yielded an excellent current efficiency of 2.35 cd A^–1^ and a maximum luminescence of 3780 cd m^–2^ with a turn-on voltage of 6.5 V, maintaining 54% of the initial luminescence at 30% strain. Regardless of the device structure, when qualifying strong luminescence, the highest possible carrier mobility could enable efficient exciton recombination towards highly efficient light-emitting devices.

## HIGH MOBILITY INTEGRATED WITH PHOTOLITHOGRAPHY PATTERNING

Facile photopatterning of high-mobility polymer semiconductors is of vital importance for fabricating multilayer functional devices and high-density integrated circuits. High photopatterning resolution, mobility retention and processing stability are critical factors for all-photolithography patterning.

Wei *et al*. developed a semiconducting photoresist involving the polymer semiconductor poly(tetrathienoacene-diketopyrrolopyrrole), a cross-linkable small-molecule monomer, a radical photo-initiator and a thiol additive for ensuring efficient photo-crosslinking [9]. The nano-interpenetrating microstructure enabled submicron resolution and dense π−π stacking, thus delivering high-density transistor arrays reaching 1.1 × 10^5^ units cm^−2^ with the highest mobility being 1.64 cm^2^ V^–1^ s^–1^. Bao *et al*. proposed the covalently embedded *in-situ* rubber matrix (iRUM) strategy to endow high-mobility conjugated polymers with additional mechanical elasticity, solvent resistance and high-precision photopatterning [10]. Leveraging the reactivity discrepancy of azide groups with C−H and C=C bonds, more reactive azide/C=C cycloaddition resulted in the polymer semiconductor network being evenly embedded into the elastic rubber matrix without disrupting semiconductor aggregation. In fully stretchable transistors, the iRUM-poly(indacenodithiophene-*co*-benzothiadiazole) film retained initial mobility at 100% strain and maintained over 1 cm^2^ V^–1^ s^–1^ after 500 stretching–releasing cycles at 50% strain, accompanied by a stable cycling life extended 5000-fold. Simultaneously, interfacial crosslinking of semiconducting and dielectric layers avoided interfacial delamination under strain, further facilitating the fabrication of fully patterned elastic transistors.

Photolithographic species generally contain dual-/multi-components with photo-reactive azide and diazirine groups attached, accompanied by puzzles like mutual miscibility, phase separation and film reproducibility. Zhang *et al*. developed a mono-component semiconducting photoresist via appending azide groups in alkyl chains of conjugated polymers [11]. Upon UV-light irradiation, transformed nitrene units crosslinked alkyl side chains towards great discrepancy in solubility and thus high-resolution photopatterning (5 μm). Patterned thin films showed an average mobility of 0.61 cm^2^ V^–1^ s^–1^ with satisfactory uniformity, and maintained π−π stacking. Unfortunately, single-component semiconducting photoresists require complex synthesis, and photo-reaction products might reside as trapping or scattering sites for carrier transport.

## HIGH MOBILITY INTEGRATED WITH EFFICIENT THERMOELECTRIC CONVERSION

The Internet of Things requires lightweight power-supplying elements, and thermoelectric materials could directly convert renewable heat sources into electricity via the Seebeck effect. High-mobility conjugated polymers are expected to break the trade-off relationship of thermoelectric parameters, delivering significantly improved electrical conductivity upon chemical doping.

Leclerc *et al*. substituted linear alkyl-chains with isometric single-ether-functionalized side chains to produce the slightly polarized poly(2,5-bis(3-dodecyl-2-thienyl)-*co*-thieno[3,2-b]thiophene) analogue with the aim of achieving enhanced cohesive forces and polymer-dopant intercalation within side-chain layers [12]. Polymer chains could be uniaxially aligned towards high dichroic ratios up to 20, and dopants were randomly distributed in amorphous side-chain regions, bringing about a unidirectionally high electrical conductivity of 5 × 10^4^ S cm^–1^ and a record power factor (PF) of 2.9 mW m^–1^ K^–2^. Limited to electron mobility, doping efficiency and ambient stability, the thermoelectric performance of n-doped materials lags far behind. Huang *et al*. developed the side-chain-free and *in-situ* reductive n-doped conducting polymer poly(benzodifurandione), which has excellent conductivity approaching 2 × 10^3^ S cm^–1^ [13]. A negatively charged conjugated backbone with ∼0.9 charges per repeating unit enabled it to have intrinsic solubility in polar solvents, and condensed films exhibited a metallic state and coherent carrier transport with PF ∼90 μW m^–1^ K^–2^. In addition to achieving balanced thermoelectric parameters towards maximum conversion efficiency, theory-guided chemical doping, intrinsic host–dopant interactions and all-level stability are problems that deserve more attention.

## OUTLOOK

Established on mutual trade-offs between electrical, mechanical, optical and thermal characteristics, high-mobility polymer semiconductors are marching towards multifunctionalization. Apart from mobility and comprehensive performance improvement, there still exist many puzzles and bottlenecks for practical application to overcome. First, a rigid coplanar backbone and strong donor–acceptor coupling contribute to superior carrier mobility, commonly accompanied by mechanical brittleness, fluorescence quenching and low-efficiency doping. Amorphous conjugated polymers with efficient intramolecular charge transport could afford unexpectedly outstanding molecular-weight-dependent functionalization. Second, electronic devices require environmental, temporal and operational stability, resulting in high demand for encapsulation layers, functional layers and layer-by-layer interfaces. Besides material selection and device technology, developing advanced orthogonal reactions and crosslinking chemistry might be feasible methods. Finally, multifunctional integration with mutual compatibility/promotion is the future of high-mobility polymer semiconductors. Additionally, expanding and fusing more special characteristics, such as magnetic, electrochemical and acoustic properties, could be equally important. Novel molecular design, doping/blending strategies and alignment solution processing all provide brand new pathways towards all-round multifunctionalization.
